# Patterns of mortality during pandemic: An example of Spanish flu pandemic of 1918

**DOI:** 10.3897/popecon.4.e53492

**Published:** 2020-04-30

**Authors:** Natalia S. Gavrilova, Leonid A. Gavrilov

**Affiliations:** University of Chicago, 60637, USA; Federal Research Institute for Health Organization and Informatics of Ministry of Health of the Russian Federation, Moscow, 127254, Russia; Institute of Socio-Political Research at the Federal Center of Theoretical and Applied Sociology of the Russian Academy of Science, Moscow, 119333, Russia

**Keywords:** mortality, infections, Spanish flu pandemic, COVID-19, J0, J1, I1

## Abstract

Now the attention of the whole world is focused on the developing pandemic of the coronavirus infection COVID-19. This article discusses mortality patterns of the deadliest epidemic in the last 120 years – the Spanish flu pandemic of 1918. Statistical sources from Italy and the USA, published shortly after the pandemic, were analyzed. The analysis was carried out for mortality from all causes, since in this case inaccuracies associated with establishing the causes of death are minimized. Despite the fact that the first cases of the Spanish flu appeared in the United States as early as March 1918, this first wave of epidemic practically did not affect the total mortality rate. The main peak of mortality in 1918 occurred in October 1918 both in the USA and Italy, with a gradual decrease in mortality over several months. Analysis of age-specific mortality demonstrates a significant increase in mortality at middle ages (20–50 years) in 1918 compared with 1917. Analysis of mortality trends using the method of latent variables shows a significant increase in the background mortality factor in 1918, which turned out to be higher for Italy than the mortality losses during the Second World War. The Spanish flu pandemic differs from the current coronavirus pandemic, because of significant increase in mortality of middle-aged people, while the COVID-19 pandemic causes a more marked increase in mortality among the elderly. With this, the COVID-19 pandemic is more like the recent flu epidemics than the earlier Spanish flu pandemic.

Currently, the world’s attention is focused on the news of the coronavirus pandemic. Right now it is difficult to assess the true extent of this pandemic, since the number of infected people depends on the number of tests performed, and the number of deaths from coronavirus depends on the specifics of cause-of-death registration in each particular country or even region. For this reason, many researchers analyzing the influenza epidemics do not analyze mortality from a specific cause, but rather mortality from all causes, since it is almost impossible to make a mistake in the fact of death. The coronavirus pandemic is not the first pandemic in human history or even the first pandemic in the last 200 years. The most famous and deadly is the 1918 Spanish flu pandemic (Viboud and Lessler 2018). In the post-war years, the 1969–70 influenza pandemic was also causing significant losses not only among the elderly (which are usually observed during regular influenza epidemics), but also at middle ages (Rizzo et al. 2007).

In this paper, we consider the patterns of mortality during the Spanish flu pandemic of 1918 using the data for Italy and the USA. These countries are currently leading in the number of persons infected with COVID-19. An analysis of mortality in 1918 and the adjacent years may help us to better understand the course and scale of the current pandemic. Statistical yearbooks published shortly after 1918 in the respective countries were used as a data source. For Italy, these are primarily Ministro di Agricultura, Industrie e Commercio (MAIC), Direzione Generale della Statistica (DirStat) (MAIC 1924, 1925a, 1925b). For the U.S. data we used Vital Statistics Rates in the United States 1900–1940 edition (Bureau of the Census 1943). The age-specific mortality rates for the Italian population are taken from the Human Mortality Database (Human Mortality Database).

First, consider the trends of mortality rate in 1916–1919 in Italy and the USA. Figure 1 shows the values of crude death rates (per 1000 population) by calendar year for Italy. You can see that mortality rose sharply in 1918, but in subsequent years it fell quite rapidly. The crude death rate in pre-war 1913 is given for comparison, because of significant war losses in Italy in 1916–1918. In 1919, mortality already returned to the pre-war values. A similar pattern is observed for mortality trends in 1916–1920 in the United States (see Figure 2). As in the case of Italy, in the United States there was a surge of mortality in 1918 and a fairly rapid drop of mortality in 1919.

However, it is well known that the Spanish flu epidemic passed in waves and the first cases of flu were recorded in the United States in March 1918, and the second deadly peak of the Spanish flu was observed in the fall of 1918. To better understand the development of the Spanish flu epidemic in 1918 and 1919, it makes sense to consider changes of mortality from all causes by month.

Monthly mortality changes in 1918 and 1919 in Italy are shown in Figure 3. It follows from the figure that the acute phase of the Spanish flu epidemic was observed in the fall of 1918 with a surge of mortality in October. By April 1919 this elevated mortality wave totally disappeared. At the same time, the spring peak of the Spanish flu epidemic in 1918 had practically no effect on the total mortality.

Figure 4 shows monthly mortality changes in Italy for 1916–1919. It follows from the figure that the Spanish flu spring wave had little effect on total mortality, which was almost the same in the first half of 1918 as in the previous two years. However, an increased number of deaths from influenza (Spanish flu) was reported in Italy in 1919 and even in 1920 (see Figure 5).

Figure 6 shows monthly mortality changes in the United States. In this figure, one can see a slight increase in mortality in the spring of 1918, which looks insignificant compared to the peak of mortality observed in October. As in the case of Italy, a decrease in mortality to normal levels occurred by April 1919. Figures 4 and 6 show that the acute phase of the pandemic with very high mortality lasted from one to two months, but a complete mortality decline to normal levels took about eight months.

Now consider age-specific mortality changes in 1917–1919. In the case of current coronavirus pandemic it is observed that older people are more likely to die from coronavirus than younger ones. This observation is not surprising. The analysis of mortality Italy in 1969–2001 demonstrated that older people die more often during regular flu epidemics (Rizzo et al. 2007). However, during the 1969–1970 pandemic the excess mortality was observed not only at older, but also at middle ages (Rizzo et al. 2007). The Spanish flu pandemic of 1918 differs from other flu epidemics by significant relative increase of mortality at middle ages. Figure 7 shows the age-specific mortality rates for Italian women in 1917–1919. The figure for Italian men is not given here due to significant war losses in 1917–1918, distorting the age-specific mortality pattern.

Figure 7 shows a significant increase in mortality between the ages of 15 and 60 years during the Spanish flu pandemic in 1918. In 1919, an increased mortality rate among middle-aged women was also observed. while after the age of 65, mortality does not change. At the same time, we cannot say that mortality at older ages was unaffected by epidemic. Figure 8 shows age-specific mortality of women after age 65 in Italy in 1917–1919. It can be seen that the Spanish flu epidemic has added an additional mortality increment in 1918 to already high mortality rate at old age.

Effect of the Spanish flu epidemic on mortality can also be analyzed using time series of mortality data with the method of latent variables (Gavrilovet al. 2017; Gavrilova and Gavrilov 2011). To this aim, we conducted a factor analysis of mortality over the period of 1900–2014. We used so-called P-technique of factor analysis when the analysis occurs across different time points or observations (values of hazard rates at different years) for ages 25 through 85. We applied factor analysis procedure with promax rotation method using the Stata, release 13 statistical package. We identified two factors capable of explaining almost 98% of the variance in the temporal changes of hazard rates. Thus, mortality evolution in Italy can be described by the following model:

$$\mu \left(x,t\right)=\alpha_{0}\left(x\right)+\alpha_{1}\left(x\right)F_{1}\left(t\right)+\alpha_{2}\left(x\right)F_{2}\left(t\right)$$

where x is age, t is time, α(*x*), α_1_(*x*), α_2_(*x*) are three sets of parameters depending on age only, while *F*_1_(*t*) and *F*_2_(*t*) are two sets of parameters depending on time only (sets of coefficients determined by factor analysis models).

By studying the variation of these factors over time, we noted that the first factor – comparable to the senescent mortality (Gavrilov and Gavrilova 1991; Willmoth 1997) and chiefly concerning the «old ages» population – remained remarkably stable over a period of 1900–1950 (see Figure 9). The second factor – comparable to the background mortality (Gavrilov and Gavrilova 1991) and observed in the «young» population – declined from the beginning of the century. Figure 9 shows the trends in background and age-related (senescent) mortality factors in Italy. In this figure, peaks of background mortality are noticeable in 1918 (the epidemic of Spanish flu) and in the 1940s (World War II). This figure shows that in Italy the effect of the Spanish flu epidemic on mortality was even stronger than the effect of hostilities during the World War II. It is also seen that subsequent influenza epidemics did not have a noticeable impact on background mortality.

The data presented here clearly demonstrate that the Spanish flu pandemic cannot be compared to the later flu epidemics and pandemics of influenza by its effect on mortality. A distinctive feature of the 1918 pandemic was a high percentage of deaths among young and middle-aged people with a relatively low increase in mortality among the elderly. Studies have shown that this phenomenon was observed among the population of Europe and the United States, but in the more remote regions of Latin America and the Pacific Islands, the mortality of the elderly from the Spanish flu was even higher than at younger ages. It is believed that exposure to influenza viruses in childhood could have a protective effect in the elderly in Europe and the USA during the 1918 pandemic (Simonsen et al. 2018).

Returning to the epidemic of coronavirus, it should be noted that there is a certain similarity in the damage to respiratory system produced by the coronavirus and the Spanish flu virus, including acute respiratory distress syndrome (Cilek et al. 2018). Right now we can do only preliminary estimates regarding the impact of the coronavirus epidemic on total mortality. The Italian Institute of Statistics (Instituto Nazionale di Statistica) regularly publishes the number of deaths from all causes for some municipalities on its website (www.istat.it). According to these data, during the period from March 1 to March 28, 2020, 29,565 people died in the studied municipalities of Italy. During the same period of 2019, 14,603 people died in the same municipalities. Thus, the number of deaths in the studied municipalities of Italy in March 2020 doubled. For comparison, the death toll in Italy in October 1918 (the peak of the epidemic) was five times higher than in October 1917. In the United States, mortality in October 1918 was 3.6 times higher than in October 1917. These data suggest that the coronavirus epidemic must be taken seriously. Yet the coronavirus epidemic has significant differences from the Spanish flu epidemic. The main difference is that the group with the highest risk of death from coronavirus is mainly elderly people with burden of chronic diseases, although deaths of healthy middle-aged adults also occur. Mortality at older ages is highly seasonal, mainly due to seasonal influenza epidemics (Rosano et al. 2019). Therefore, the effect of the coronavirus pandemic on mortality can be better assessed by studying the entire set of data on seasonal mortality, including data for 2020, which are currently insufficient. One can only assume that the coronavirus epidemic is likely to lead to a parallel upward shift in mortality for ages over 20 years (in semi-log coordinates), similar to what happened during the Spanish flu epidemic for ages over 60 years (Fig. 8). Some optimism is inspired by the fact that the acute period of the Spanish flu epidemic with unusually high mortality did not last long – one to two months. It should be remembered that the waves of the epidemic continued after 1918 until 1920, but their impact on the total mortality was negligible. Therefore, we can hope that in the case of coronavirus pandemic, the acute phase heralded by mortality surge will not last long.

## Figures and Tables

**Figure 1.**
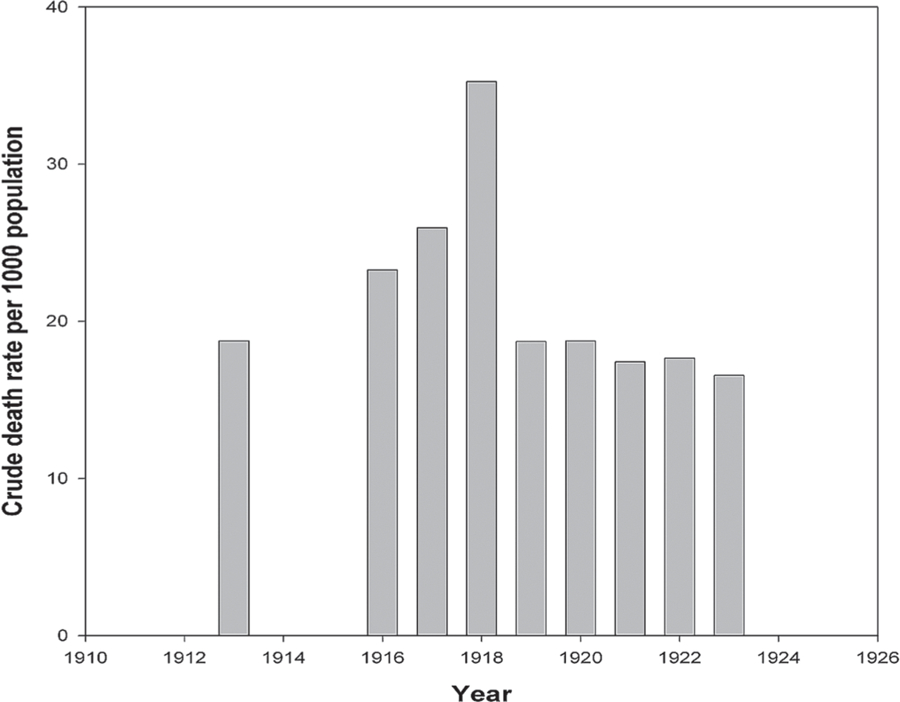
Crude death rate (per 1000 population), by calendar year in Italy. **Source:** MAIC 1924, 1925a, 1925b

**Figure 2.**
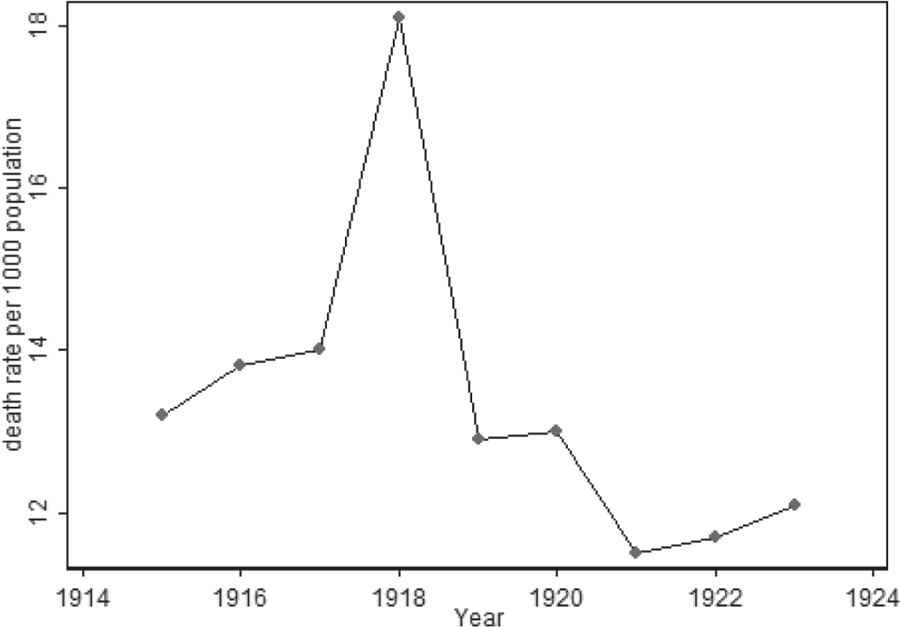
Crude death rate (per 1000 population), by calendar year in the United States. **Source:** Bureau of the Census 1943

**Figure 3.**
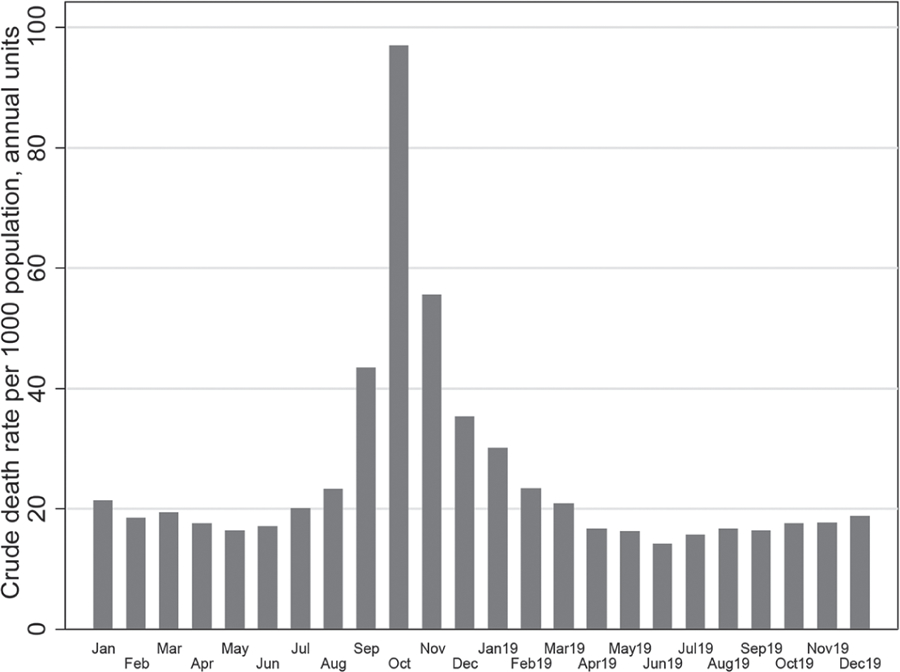
Crude death rates by month (per 1000 population) in Italy starting from January 1918. Rates computed on an annual basis. **Source:** authors’ estimations based on (MAIC 1924, 1925a, 1925b)

**Figure 4.**
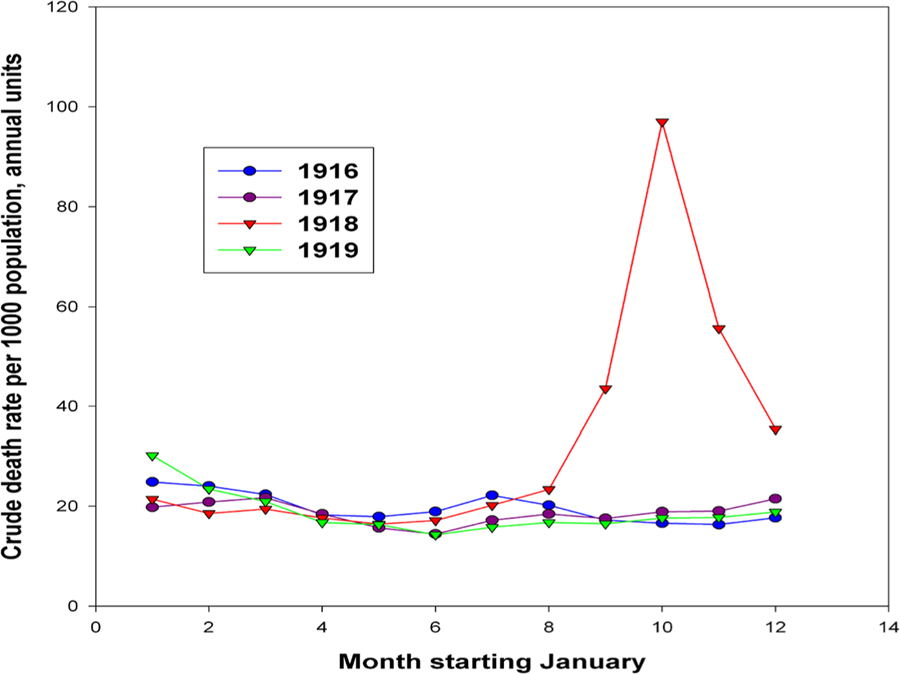
Crude death rates by month (per 1000 population) in Italy in 1916–1919. Rates computed on an annual basis. **Source:** authors’ estimations based on (MAIC 1924, 1925a, 1925b)

**Figure 5.**
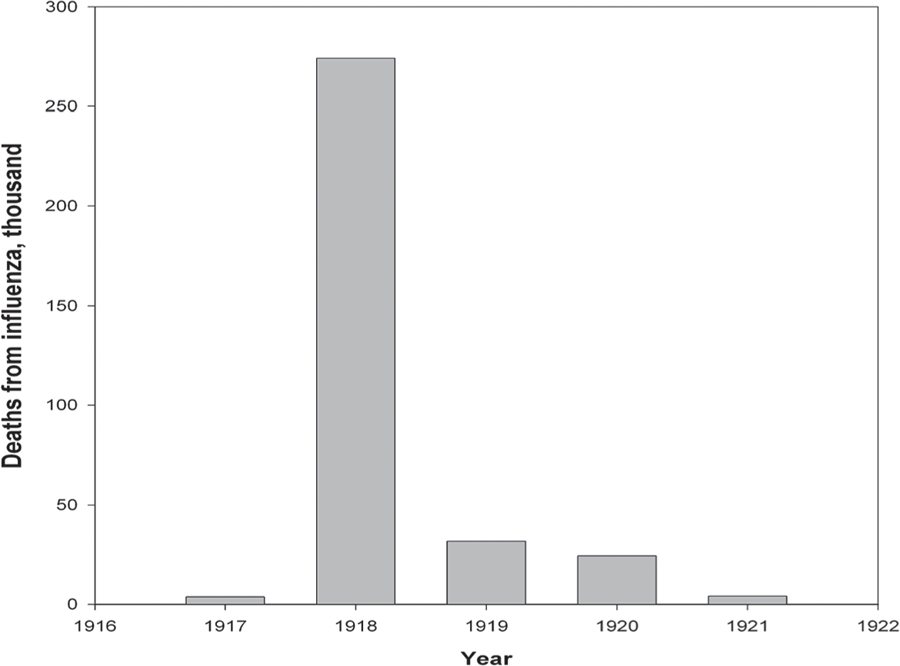
Deaths from influenza in Italy (in thousand), by calendar year. **Source:** MAIC 1924, 1925a, 1925b

**Figure 6.**
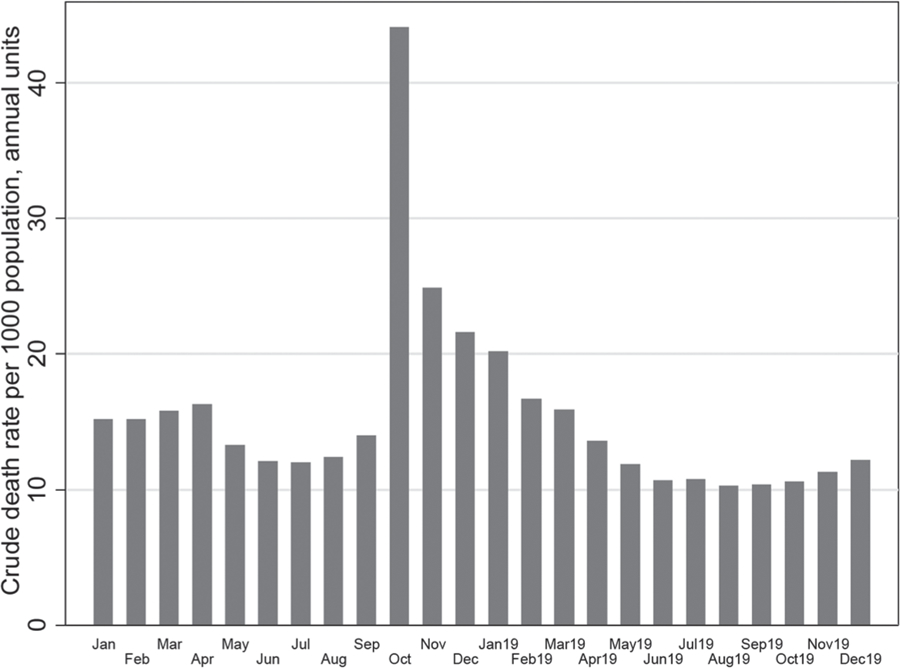
Crude death rates by month (per 1000 population) in the United States in 1918–1919. Rates computed on an annual basis. **Source:** authors’ estimations based on (Bureau of the Census 1943)

**Figure 7.**
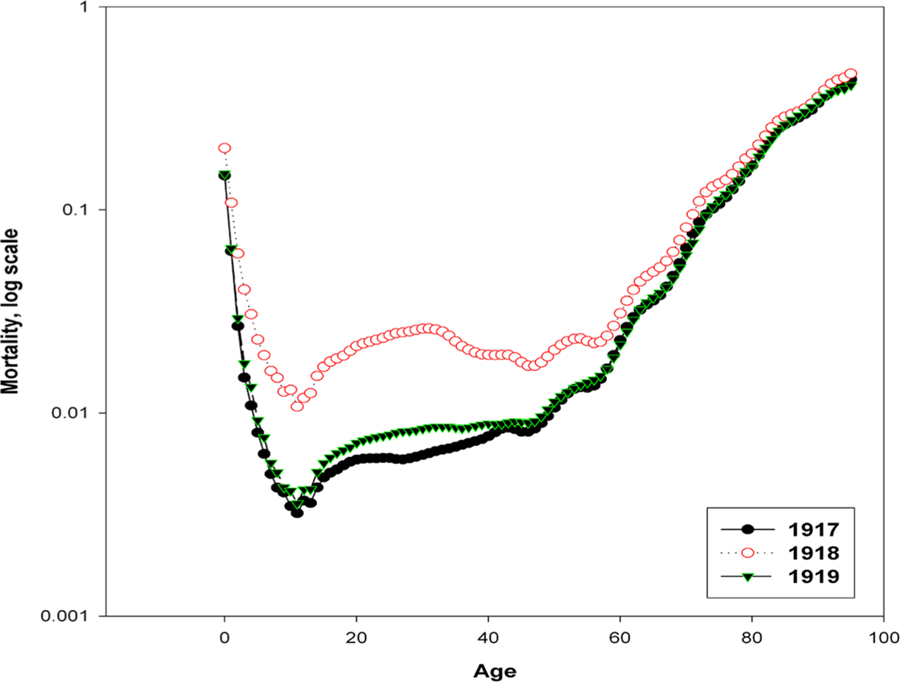
Age-specific death rates for Italian women in 1917, 1918 and 1919. **Source:** Human Mortality Database

**Figure 8.**
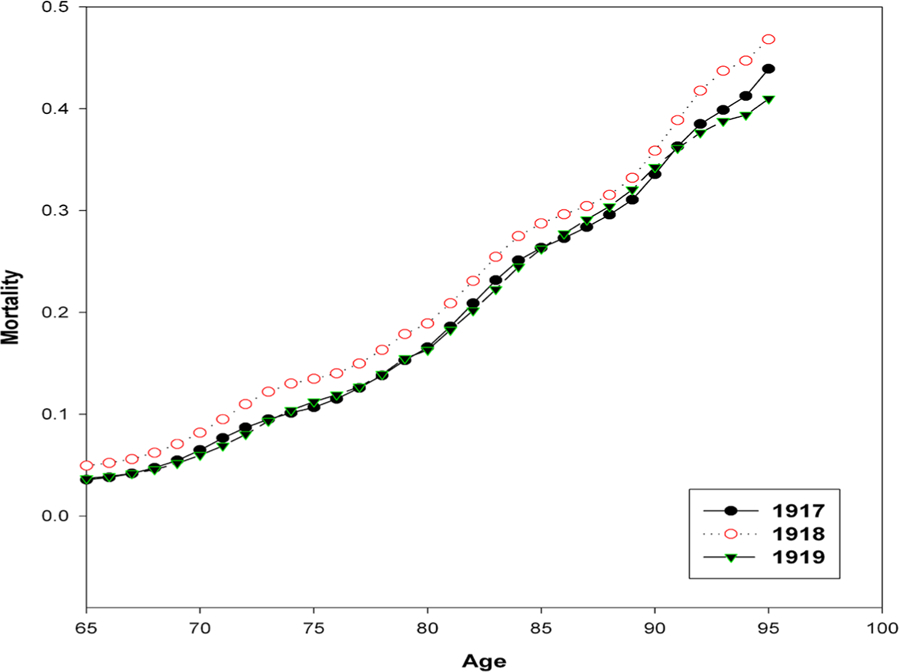
Age-specific death rates for Italian women after age 65 in 1917, 1918 and 1919. **Source:** Human Mortality Database

**Figure 9.**
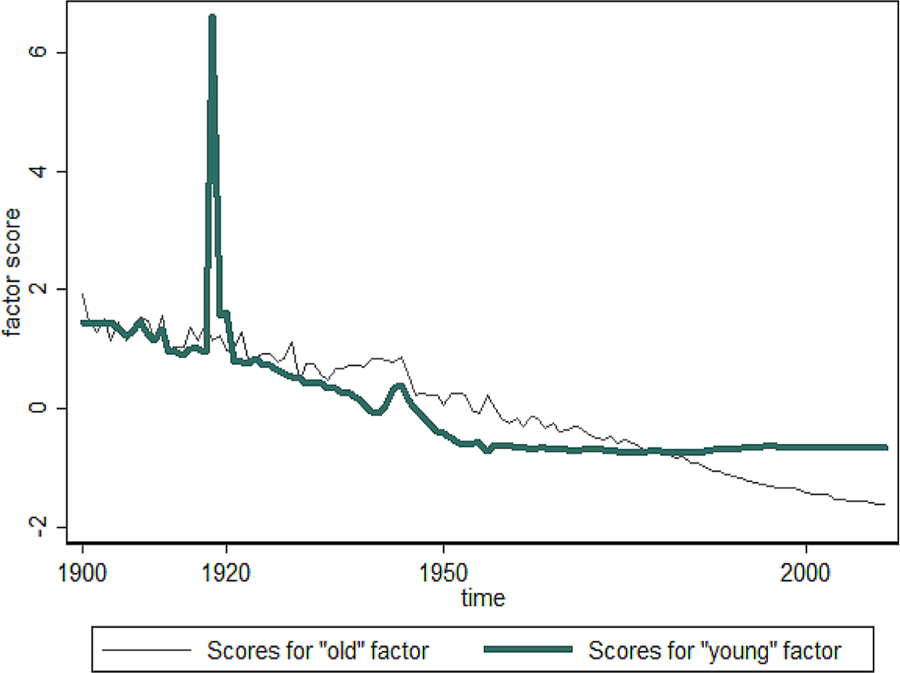
Senescent («old») and background («young») factors of mortality for Italian women. **Source:** authors’ estimations
